# Emerging drug delivery approach using nanomaterials for the treatment of endometrial cancer

**DOI:** 10.3389/fmedt.2025.1680519

**Published:** 2025-11-28

**Authors:** Zhuorong Miao, Xiaoyan Xiong, Jiahong Gao, Yubing Hu, Dongdong Jin, Guiyuan Yu, Ping Jin, Wenjun Chen

**Affiliations:** 1 Shenzhen Maternity and Child Healthcare Hospital, Women and Children’s Medical Center, Southern Medical University, Shenzhen, Guangdong Province, China; 2 Sauvage Laboratory for Smart Materials, School of Integrated Circuits, Harbin Institute of Technology (Shenzhen), Shenzhen, China

**Keywords:** nanomaterials, endometrial cancer, drug delivery, nanoplatform, fertility-preservation treatment

## Abstract

Endometrial cancer, accounting for over 90% of uterine malignancies, has experienced a significant global rise in incidence and mortality. Conventional therapies face limitations including fertility compromise, systemic toxicity, drug resistance, and poor outcomes in advanced/recurrent cases. Considering the unique physical and chemical properties of nanomaterials, the emerging drug delivery approaches based on nanomaterials are regarded as a promising pathway for enhanced therapeutic efficiency to combat endometrial cancer. Herein, this mini-review discusses emerging drug delivery approaches to overcome current treatment challenges. We classify common therapeutic nanomaterials into polymer-based nanocarriers, quantum dots, liposomes, and exosomes, analyzing their synthesis, mechanisms, and preclinical efficacy. Finally, scientific challenges and future perspectives for ongoing research in this field are presented.

## 1 Introduction

Endometrial cancer (EC) is the sixth most common malignancies of the female reproductive system (1, 2). With changes in lifestyle and environmental factors, both the incidence and mortality of endometrial cancer continue to increas (3). For early-stage disease, the main treatment is surgery, such as the hysterectomy, but young patients need to face with losing fertility forever (4, 5). Moreover, depending on the specific disease stage and other risk factors, adjuvant radiotherapy and/or chemotherapy can be used to reduce recurrence risk (2, 6). Chemotherapy and radiotherapy usually face the poor tumor specificity, systemic toxicity, and adverse effects such as bone marrow suppression and hepatic/renal damage. To address the challenges and limitations associated with conventional therapies, researchers have increasingly focused on nanomaterials. These advanced approaches aim to improve therapeutic efficacy by enhancing tumor specificity, reducing systemic toxicity, and enabling targeted drug delivery, thereby offering promising alternatives for the treatment of endometrial cancer, especially for the women in their reproductive years. And it is necessary to summarize the current progress to understand the challenges and opportunities of nanomaterials-based drug delivery approaches in EC fertility-sparing management.

This review provides a comprehensive discussion of various nanomaterials in the treatment of endometrial cancer, classified into polymer-based nanocarriers, quantum dots, liposomes, exosomes, and other nanoparticles. We first introduce the concepts, classification, and current treatment modalities of endometrial cancer. Then, the synthesis, advantages and applications in EC treatment of each type of nanomaterials are discussed. A conclusion and perspective are provided in the end. Overall, our review aims to offer an overview of current achievements and challenges to inspire the future directions of this field.

## 2 Fundamentals and pathological classification of endometrial cancer

**Figure 1:**
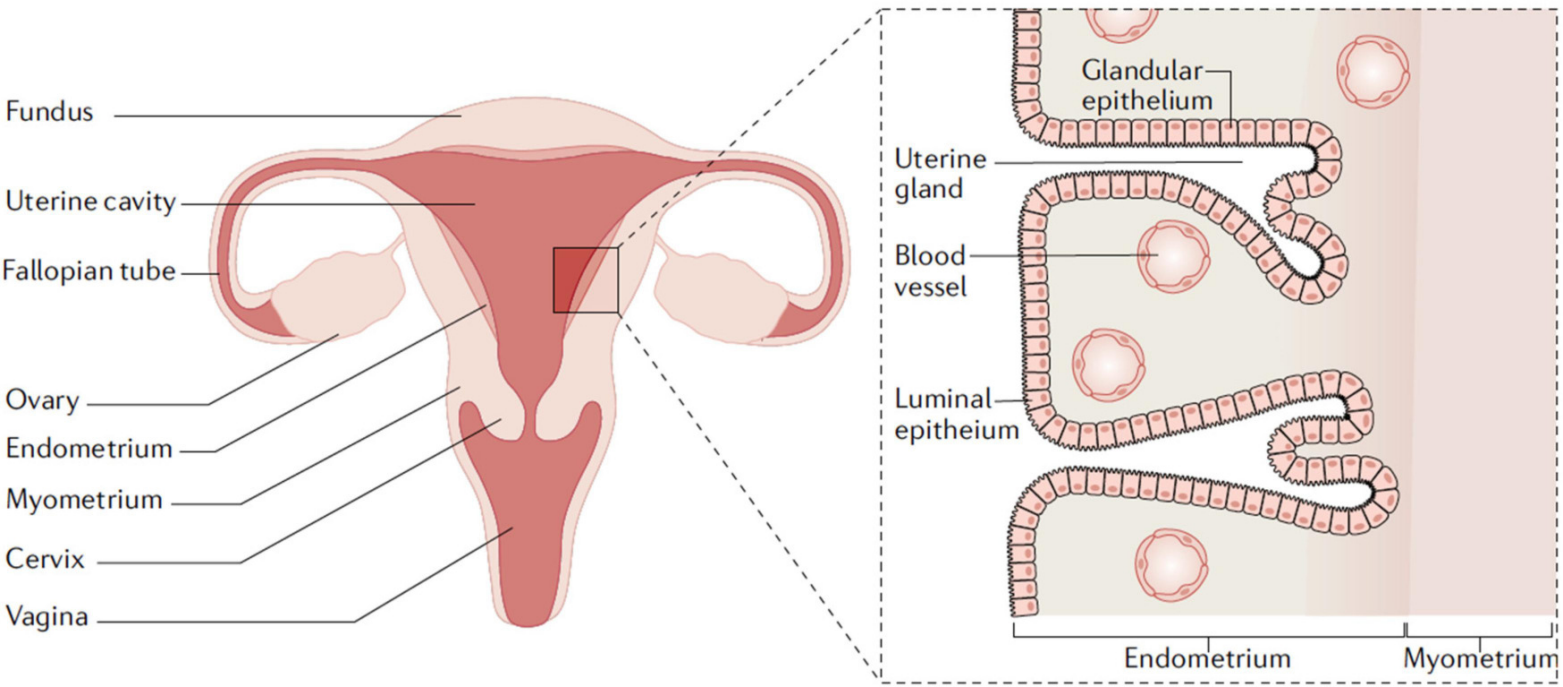
Uterine anatomy. The endometrium is the inner lining of the uterus. Endometrial cancer arises from the endometrial glandular epithelium. Adapted with permission (2). Copyright 2021, Nature Reviews Disease Primers.

Endometrial cancer (EC) is a malignancy of the inner epithelial lining of the uterus (2) (Figure 1). It primarily affects postmenopausal women and is often associated with prolonged exposure to estrogen or gene mutations, such as those in PTEN, POLE and TP53 (7, 8). Additional risk factors include obesity, diabetes, and hypertension (9). Based on the pathogenesis and histopathological characteristics, endometrial cancer is classified into two main types: estrogen-dependent type (Type I) and non-estrogen-dependent type (type II). Type I endometrial cancer is more prevalent and typically presents as endometrioid carcinoma. It tends to occur in young patients, is generally well differentiated, and exhibits high positivity for estrogen and progesterone receptors, resulting in a more favorable prognosis (10). In contrast, type II endometrial cancer is not clearly associated with estrogen exposure. It includes histological subtypes such as serous carcinoma, clear cell carcinoma, carcinosarcoma, all of which are rare, poorly differentiated, and highly aggressive. The subtypes often exhibit negative or low expression of hormone receptors and are associated with a poor prognosis (11). In addition, rare variants such as mixed-type carcinomas and neuroendocrine tumors may also occur.

EC has a relatively high early diagnosis rate, contributing to a generally favorable prognosis. However, once the cancer metastasizes, the five-year survival rate decreases significantly. Patients usually have symptoms such as vaginal bleeding, abdominal pain or abnormal vaginal discharge (12). In advanced stages, the endometrial cancer may invade the myometrium, cervix, or metastasize to distant organs. In severe cases, it can lead to organ failure and even death. While traditional treatment modalities, such as surgery and radiotherapy, have improved patient outcomes, many patients of reproductive age lose their fertility due to the interventions. Patients who receive conservative treatments face a relatively high risk of recurrence, and the survival rates for those with advanced or recurrent cancer remain low (13). Furthermore, the psychological pressure stemming from intensive treatment and the uncertain prognoses can affect patients' well-being. Therefore, early screening for endometrial cancer is essential, and there is a pressing need to develop novel fertility-sparing strategies for the management of endometrial cancer.

## 3 Current treatment modalities, limitations, and challenges in endometrial cancer

The treatment of endometrial cancer is mainly determined by the disease stage. The main therapeutic approaches include surgical intervention, radiotherapy, chemotherapy, hormonal therapy, and immunotherapy.

For the early-stage EC, the main treatment is surgery. Total hysterectomy with bilateral salpingo-oophorectomy is the primary treatment (13). In some cases, pelvic lymph node dissection or sentinel node biopsy may be added but the risks like lymphedema may increase. The adjuvant radiotherapy and/or chemotherapy can be used to reduce risk of recurrence after surgery (14). Radiotherapy can reduce the risk of vaginal and pelvic recurrence (15). However, it may cause associated side effects, such as radiation-induced cystitis. Chemotherapy is crucial for advanced or recurrent disease, inhibiting tumor growth and enhancing immune response when combined with checkpoint inhibitors. Taxane-platinum combinations show high response rates (67%) (3), but drug resistance remains a major challenge. In addition, management of EC in young women who desire to maintain fertility presents a unique set of challenges since the standard surgical treatment is not compatible with the patient's goals (5).

14% EC are diagnosed in premenopausal women, with 5% occurring in women under 40 years (16). These young patients are typically nulliparous and often present at an early stage who wish to preserve their fertility. This creates a profound conflict between oncologic treatment and reproductive goals. The standard of care for EC is total hysterectomy, which results in irreversible infertility. Thus, fertility-sparing treatment, primarily using hormone therapy, has emerged as an option for young female (2, 17). Hormonal therapy is especially crucial infertility-preserving treatments and as a non-surgical option for advanced cases. This approach usually requires the administration of oral or intrauterine progestins, to induce a complete response and create a window for childbearing prior to the required standard surgery (18, 19). Progesterone therapy is effective in patients with well-differentiated endometrioid carcinoma who are estrogen/progesterone receptor positive. However, the application is limited to receptor-positive patients, and long-term use may lead to side effect such as thrombosis and weight gain (13). Overall, current fertility-preserving treatments for early-stage endometrial cancer, for young patients who want to preserve fertility, are constrained by limitations and challenges. Strict patient eligibility criteria restrict this option to only those with low-grade, non-invasive disease, yet pre-operative imaging often fails to definitively exclude more advanced pathology. Even for ideal candidates, treatment efficacy is not guaranteed, with inherent progestin resistance leading to incomplete response in a substantial minority of patients. For those who do achieve a complete remission, the risk of recurrence is high, necessitating invasive and frequent surveillance biopsies. Furthermore, successful treatment does not equal to reproductive success, as patients often face underlying infertility comorbidities and a potentially compromised endometrial environment, requiring complex assisted reproductive technologies to achieve a live birth before ultimately undergoing definitive surgery.

More recently, immunotherapy alone or in combination has also become standard of care, although these therapies are not universally available across all jurisdictions (20). Some studies have evaluated the activity of checkpoint inhibitors in EC. For instance, combining PD-1 inhibitors with anti-angiogenic drugs has proven effective in the treatment of pretreated recurrent EC (21). Besides novel therapies such as immunotherapy, innovative therapeutics combining nanomaterials with chemotherapeutic drugs may also provide a new direction to improve survival while minimizing adverse effects and preserving fertility.

## 4 Nanomaterials-based strategies

Drug resistance and immune escape are two major barriers in the treatment of cancers. Tumor cells employ diverse mechanisms to develop resistance to chemotherapeutic agents and evade immune surveillance, leading to treatment failure and malignant progression of tumors (22, 23). Nanomaterials-based drug delivery systems show significant potential in cancer therapy due to their unique physicochemical properties and multi-functional integration capabilities. Their small size enables deep tumor penetration and passive targeting via the enhanced permeability and retention (EPR) effect (24), while surface functionalization allows for active targeting of specific cancer cell markers (25). Additionally, their high surface area-to-volume ratio facilitates efficient drug or gene loading and controlled release (26). Thus, through precise targeting, stimulus response release, and multi-functional treatment can enhance the therapeutic efficacy and minimize the systemic toxicity, providing complementary approaches to endometrial cancer treatment.

Here, we summarized the applications of various nanomaterials, including polymers, liposomes, exosomes, quantum dots, etc. in endometrial cancer treatment.

### 4.1 Polymer-based nanocarriers

Polymer-based delivery carriers have been well studied in drug delivery (27), gene therapy (28), and vaccine development (29) owing to their design flexibility, stability, functional versatility, and relatively low production costs. Their high drug-loading capacity further positions them as promising tools for endometrial cancer treatment.

**Figure 2:**
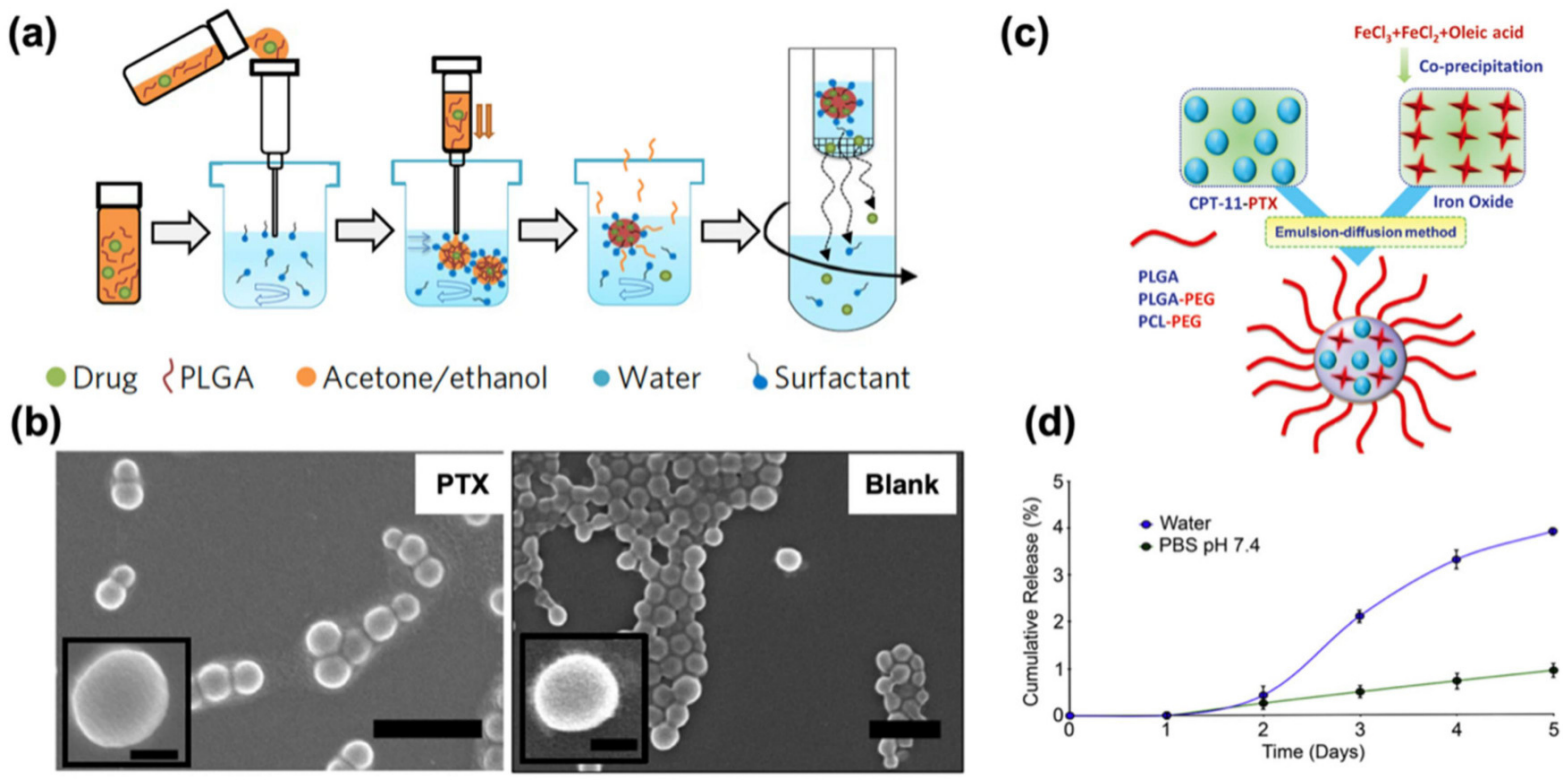
**(a)** Schematic illustrating the nanoprecipitation method used for nanoparticle preparation. Adapted with permission (33). Copyright 2017, Nature Nanotechnology. **(b)** Scanning electron micrographs of PTX (Left) and Blank (Right) showing spherically shaped nanoparticles with smooth surfaces. Scale bar = 500 nm (100 nm in the insert). Adapted with permission (33). Copyright 2017, Nature Nanotechnology. **(c)** Schematic representation of SPIO/PTX-loaded PLGA-based nanoparticles. Adapted with permission (34). Copyright 2021, Materials Express. **(d)** Time course in days demonstrating the cumulative release of paclitaxel from 5 mM-loaded nanoparticles in the presence of water (blue line) or PBS, pH 7.4 (green line). Adapted with permission (35). Copyright 2013, Biomaterials.

Poly (lactic-co-glycolic acid) (PLGA)-based nanoparticles (NPs) are widely investigated as drug delivery systems. Approved by both the U.S. Food and Drug Administration (FDA) and the European Medicines Agency (EMA), PLGA is a polymeric organic compound known for its excellent biocompatibility, biodegradability, and favorable physicochemical properties (30–32). These characteristics make PLGA one of the most widely used and effective polymers for drug delivery applications. Ebeid et al. (33) established that paclitaxel (PTX)-loaded PLGA nanoparticles showed enhanced therapeutic efficacy compared with pure PTX. PTX-loaded NPs were prepared through a nanoprecipitation method, resulting in diameter <175 nm with smooth surfaces (Figure 2a). And loading PTX into the NPs did not affect the integrity or surface morphology when compared to Blank, as shown in Figure 2b. PTX-loaded PLGA NPs delivery system achieved synthetic lethal therapy for endometrial cancer with improved efficacy due to enhanced dissolution, improved pharmacokinetics, and minimized side effects, as well as the enhanced permeability and retention (EPR) effect of nanoparticles. *In vivo* experiments have shown that this polymer can inhibit the progression of endometrial cancer, prolong the survival period of patients with good safety.

In addition, in view of the limitations in the treatment of endometrial cancer, in order to explore efficient and safe treatment methods, Chen et al. (34) prepared a dual-loaded nanopolymer for therapeutic diagnosis, which was made of paclitaxel (PTX) and superparamagnetic iron oxide (SPIO) loaded PLGA (Figure 2c). The polymer is spherical and has a size of approximately 240 nm. The loading amounts of PTX and iron were 1.84 ± 0.4 and 10.4 ± 1.93 mg/100 mg, respectively. It can significantly increase the uptake of Ishikawa cells while inhibiting the growth of Ishikawa cells. Meanwhile, due to its high cell uptake rate and magnetic characteristics, it can be used as a potential tumor-targeted MRI contrast agent and is expected to become a nanomedicine for imaging, drug delivery and real-time monitoring of therapeutic responses.

Melendeza et al. (36) prepared an intrauterine Everolimus drug delivery system based on poly (propylene glycol fumarate) (PPF)/N-vinylpyrrolidone (NVP) polymer. This polymer is biocompatible and showed no obvious inflammation in the rat uterus. *In vivo* experiments demonstrated that Everolimus rods can continuously deliver the drug locally to the uterus for 84 days, with low systemic exposure. Everolimus in the uterus has biological activity and can inhibit the PI3K/AKT/mTOR pathway and cell proliferation. This strategy is expected to be used for non-surgical treatment of patients with atypical hyperplasia and early low-grade endometrial cancer.

In terms of exploring the interaction and release mechanism between drugs and polymers, studies suggest that poly (3-hydroxybutyric acid—co-3-hydroxyvaleric acid) (PHBV) nanoparticles have considerable potential in large-scale drug production. Because PHBV is a biodegradable, non-toxic, and low-cost polyester, it is often used in tissue engineering and drug delivery. Based on the characteristics of PHBV and the wide application of paclitaxel, Vilos et al. (35) prepared PHBV nanoparticles loaded with paclitaxel by the modified double emulsion solvent evaporation method. The average diameter of the nanoparticles was 228–264 nm, the drug loading was proportional to the initial drug concentration, and the encapsulation efficiency was approximately 37%. The surface of nanoparticles has pores, which may delay drug release (Figure 2d). They can be taken up by cancer cells and have high cytotoxicity to endometrial cancer cells and primary ovarian cancer cells. They have a low production cost and are strong candidates for new drug-loaded nanoparticles.

Moreover, in order to find an effective treatment for type II endometrial cancer and reduce the side effects of paclitaxel (PTX), Naguib et al. (37) developed a novel ciprofloxacin derivative loaded with polyethylene glycol polymer nanoparticles (CIP2b-NPs), which was used in combination with paclitaxel for the treatment of human type II endometrial cancer. The results confirmed that the polymer nanoparticles were spherical with a particle size of 151.6 nm, and the drug loading was approximately 881 *μ*g/mL. They were released slowly *in vitro* and were more easily uptaken by cells, enhancing the cytotoxicity of paclitaxel and the G2/M phase arrest effect. Meanwhile, the synergistic application of paclitaxel and CIP2b nanoparticles can significantly inhibit the growth of endometrial cancer and has good safety. In addition, given the upregulation of histone deacetylase (HDAC) in endometrial cancer and the poor efficacy of the traditionally used HDAC inhibitor SAHA in the treatment of endometrial cancer, Edwards et al. (38) synthesized F127 polymer micelles with hyaluronic acid on the surface to encapsulate SAHA to improve the efficacy and targeting. And they evaluated the therapeutic effect in 2D and 3D models. The results confirmed that this nano-system enhanced the delivery and activity of SAHA, improved the internalization ability of the nanoparticles and the penetration ability of the spheres, inhibited the growth of endometrial cancer cells, and solved the phenotypic problems that occurred when free drugs were used to treat type II endometrial cancer cells, thereby improving the therapeutic effect. In Table 1, we provide a summary of the current polymer-based strategies applied in endometrial cancer treatment.

**Table 1:** Various polymer-based systems for EC treatment.

Materials	Function	Targeting	Synthesis method	Clinical application	Targeted cells	Limitations
PTX/SPIO/PLGA	Chemotherapy drug delivery/magnetic resonance imaging	Combine magnetic targeting/surface-modified targeting	Double-loaded drug packaging	Tumor imaging and chemotherapy	Ishikawa cells	Material degradation is uncontrollable Dual-drug loading challenge Difficulty in clinical transformation
PTX/BIBF/PHBV	Dual-drug synergistic anti-cancer	Target cancer cells with LOF p53 mutations	Nano-precipitation method	Tumor synthesis can cause death and chemotherapy	Hec50co cells	Insufficient targeting Difficulty in tumor penetration Potential carrier toxicity@4.Complex production processes
Everolimus/PPF/NVP	Targeted drug delivery	Target mTOR	Cross-linking method	Targeted therapy for cancer	Intrauterine delivery	The risk of sudden drug release The problem of polymer degradation Local and systemic toxicity The applicable cancer types are limited
PTX/PHBV	Delivery of chemotherapy drugs	Passive targeting (EPR)	Double emulsion solvent evaporation method	Cancer treatment	Ishikawa cells	Lack of active targeting Carrier degradation and release control issues Insufficient *in vivo* research.
PTX+CIP2b	Dual-drug synergistic anti-cancer	Passive targeting (EPR)	Nano-precipitation method	Targeted therapy for cancer	Hec50co cells	Lack of *in vivo* data Limited targeting Preparation and transformation challenges
Thiolated HA (HA-SH)	Delivery of anti-tumor targeted drugs	Active targeting	Emulsification method, physical encapsulation	Targeted therapy for cancer	Ishikawa and Hec50 cells	Deep penetration of tumors is facing challenges Long-term security issues Difficulties in clinical transformation

Polymers-based nanocarriers with diverse functions have contributed significantly to drug delivery for EC treatment. The above-mentioned drug delivery system represents an active exploration with different focuses in enhancing tumor targeted therapy and imaging. Their advantages are reflected in overcoming the limitations of traditional therapies through nanotechnology, such as achieving passive targeting by utilizing the EPR effect, or achieving active targeting through surface modifications (such as the HA-SH system) and magnetic guidance, aiming to increase the drug concentration at the tumor site and reduce systemic exposure. Moreover, by using dual-drug combination strategies (such as PTX/BIBF, PTX+CIP2b) to enhance the anti-cancer efficacy through synergistic effects, especially for therapies targeting specific gene mutations (such as p53 LOF), the potential of precision medicine has been demonstrated. However, these systems still face some common and individual challenges. First, insufficient targeting efficiency is a common bottleneck. It limits the drug's ability to kill core tumor cells. Second, the controllable degradation of carrier materials and drug release are major challenges. Multiple systems (such as PLGA and PHBV systems) are confronted with the problem of sudden release risk or unsatisfactory release kinetics caused by uncontrollable degradation rates, which directly affects the efficacy and safety. Last, it is difficult to achieve clinical transformation. This includes the difficulty in scaling up complex production processes, the lack of sufficient *in vivo* efficacy and safety evaluation data (such as the PTX+CIP2b system), and the long-term toxicity issues that may be caused by the carrier materials themselves. In addition, there are also significant technical challenges in precisely controlling the loading, proportion and release timing of the two drugs when they are co-loaded (such as in PTX/SPIO/PLGA systems). Therefore, the development of next-generation delivery systems should focus on designing more intelligent responsive materials to achieve on-demand and controllable drug release, developing multi-modal collaborative targeting strategies to overcome tumor penetration barriers, and systematically conducting preclinical studies from *in vitro* to *in vivo*. Through comprehensive evaluation of the biocompatibility, pharmacokinetics, and ultimate efficacy of the drug delivery system. It will lay a solid foundation for genuine clinical application.

### 4.2 Quantum dots

**Figure 3:**
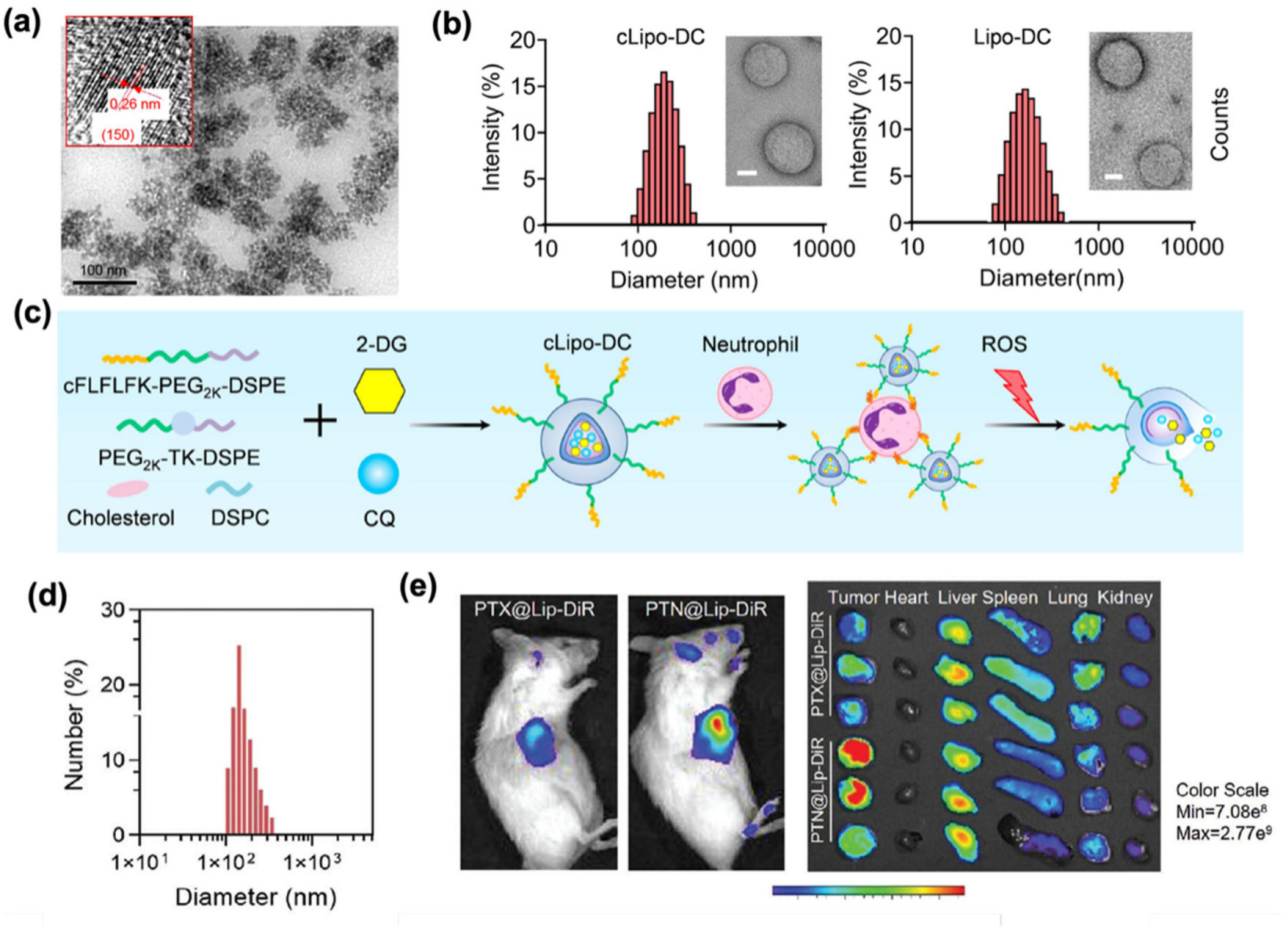
**(a)** TEM and HRTEM images (inset) of the above RQDs, with the arrows emphasizing the 0.31 nm distance of parallel between them. Adapted with permission (39). Copyright 2015, International Journal of Nanomedicine. **(b)** TEM images of cLipo-DC and its size distribution. Scale bar: 50 nm. (Left). TEM images of Lipo-DC and its size distribution. Scale bar: 50 nm. (Right). Adapted with permission (41). Copyright 2025, Theranostics. **(c)** Liposomes were synthesized using cholesterol, DSPC, ROS-responsive polymeric DSPE-TK-PEG2000, and DSPE-PEG2000-cFLFLF, with cFLFLF peptides facilitating specific binding to neutrophils through interaction with formyl peptide receptors (FPRs). 2-DG and CQ were co-loaded into liposomes to form cLipo-DC. Adapted with permission (41). Copyright 2025, Theranostics. **(d)** DLS measurement of PTN @Lip.Scale bar: 200 nm. Adapted with permission (42). Copyright 2024, Advanced Functional Materials. **(e)** Fluorescence imaging of representative U14 tumor-bearing mice intravenously injected with DiR-labeled PTX@Lip and PTN@Lip and their main organ and tumors *in vitro* recorded using an *in vivo* IVIS Spectrum Imaging System (*n* = 3). Adapted with permission (42). Copyright 2024, Advanced Functional Materials.

Quantum dots, as a type of semiconductor crystal with unique optical properties, have high quantum yield and excellent photostability, and are often used as nanoprobes to play a role in tumor diagnosis and treatment. Wang et al. (39) reported a water-soluble realgar (As_4_S_4_) quantum dots (RQDs) and demonstrated their ability to inhibit proliferation. They synthesized well-dispersed RQDs with an average size of 5.48 ± 1.09 nm and a size distribution of ∼20%, especially showing improved water solubility and bioavailability compared with traditional realgar medicines (Figure 3a). RQDs induced vacuolization and endoplasmic reticulum dilation in endometrial cancer cells, which further leaded to cell apoptosis and necrosis. Realgar quantum dots not only have the potential to fight cancer but also play an important role in the influence on autophagy of cancer cells. As Liu et al. (40) reported that RQDs induced cell cycle arrest in the G2 and S phases, promoted autophagy, and altered the expression of autophagy-related proteins, suggesting their role as autophagy inducers. In essence, QDs form transforms bulk arsenic sulfide (As_4_S_4_), which is a material traditionally limited by poor solubility and biocompatibility, into a new generation of therapeutics. It leveraged nanoscale properties such as reduced size, increased surface area, and enhanced purity, along with advanced surface engineering. These modifications result in significantly improved pharmaceutical performance and broaden the potential clinical applications of arsenic sulfide-based treatments.

### 4.3 Liposome

While quantum dots face challenges such as rapid systemic clearance, liposomes demonstrate superior biocompatibility and safety owing to their composition of phospholipids and cholesterol, which are similar to the components of cell membranes. The biomimetic structure enables liposomes to achieve versatile encapsulation of hydrophilic, hydrophobic, amphiphilic, nucleic acid, and protein therapeutics. Liposomes enhance pharmacokinetics by prolonging circulation time and facilitating targeted delivery. The low intrinsic toxicity and capacity for sustained drug release make them a crucial role in modern delivery systems, particularly in precision medicine and biotechnology. Notably, liposomal platforms show emerging promise in targeted therapies for endometrial cancer. For instance, Zhou et al. (41) designed ROS-responsive liposomes (cLipo) with neutrophil-targeted peptides modified on the surface. The glucose analogue 2-deoxy-D-glucose (2-DG) and the autophagy inhibitor chloroquine (CQ) were co-loaded into liposomes to form cLipo-DC. cLipo-DC was uniformly spherical, with an average size of 201.8 ± 7.81 nm respectively (Figure 3b), showing a zeta potential of −21.51 mV. *in vitro* experiments confirmed that it could effectively inhibit glycolysis and autophagy in endometrial cancer Ishikawa cells, thereby promoting cell death. The mechanism is shown in Figure 3c. Furthermore, to address the issues of energy dissipation and long treatment duration associated with high-intensity focused ultrasound (HIFU) for gynecologic tumors, Zhang et al. (42) developed a liposome loaded with ammonium bicarbonate and paclitaxel (PTX) (PTN@Lip) to explore methods that could enhance HIFU imaging and treatment efficacy. These liposomes exhibited an average diameter of about 140 nm (Figure 3d), colloidal stability, and pH-responsive drug release properties. In both U14 and KLE tumor models, the PTN@Lip/HIFU strategy enhanced HIFU imaging, regulated tumor microenvironment, and increased PTX uptake (Figure 3e). This combination inhibited tumor growth and activated the immune system. This strategy is expected to solve existing clinical treatment challenges, open up new directions for gynecological tumor treatment, and the ingredients used have been approved by the FDA. It represents a translatable strategy with potential for clinical adoption.

Although liposomes are widely used as drug delivery carriers, they need to face the inherent limitations that impede the further development, including oxidation of phospholipid bilayer, premature leakage of hydrophilic payloads, and the high manufacturing cost. Research focuses on engineering advanced liposomal formulation, such as polymer-stabilized, hybrid, or stimuli-responsive architectures, to address these limitations. Development in lyophilization techniques and continuous manufacturing also show promise for enhancing stability and scalability. Addressing these challenges remains critical for unlocking the full translational potential of liposome-based therapeutics in precision oncology.

### 4.4 Exosome

**Figure 4:**
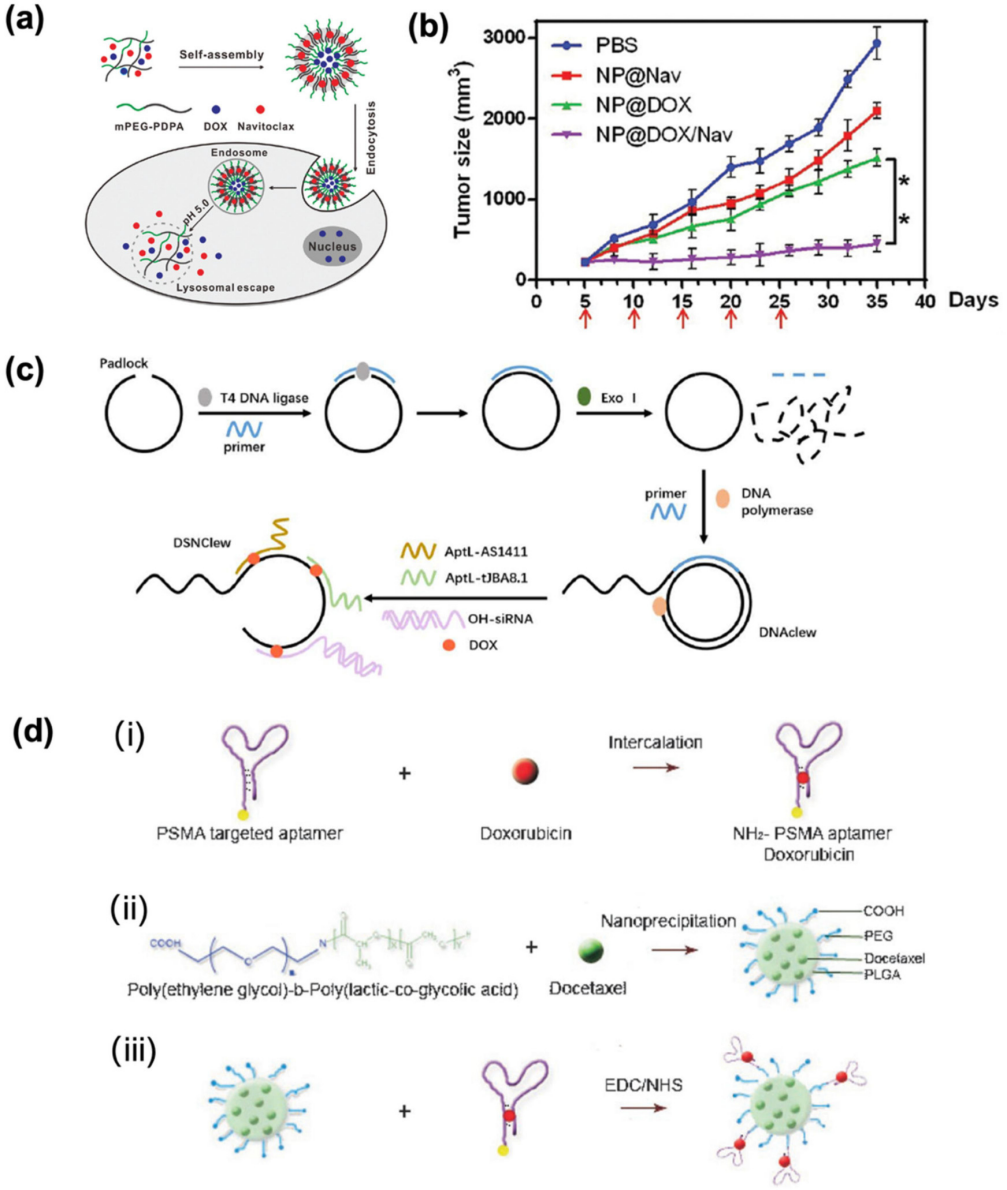
**(a)** Co-delivery of DOX and navitoclax mediated by ultra pH-sensitive polymeric nanovesicles for synergetic therapy of endometrial carcinoma. Adapted with permission (44). Copyright 2020, Biomaterials Science. **(b)** Tumor volumes change curve. Adapted with permission (44). Copyright 2020, Biomaterials Science. **(c)** DSNClew synthesis schematic. Adapted with permission (47). Copyright 2024, International Journal of Biological Sciences. **(d)** (i) the intercalation of a hydrophilic anthracycline drug, such as Dox within the A10 PSMA aptamer; (ii) the encapsulation of a hydrophobic drug, such as Dtxl, within the PLGA-b-PEG nanoparticles using the nanoprecipitation method; and (iii) nanoparticle–aptamer (NP–Apt) bioconjugates comprised of PLGA-b-PEG nanoparticles surface functionalized with the A10 PSMA aptamer for co-delivery of Dtxl and Dox. Both drugs can be released from the bioconjugates over time. Adapted with permission (48). Copyright 2007, ChemMedChem.

As endogenous nanovesicles secreted by cells, exosomes exhibit intrinsic biocompatibility and biomolecular composition mirroring human physiology. Such properties enable exosomes to evade immune clearance and cross the biological barrier. Their natural tissue-targeting specificity and homing mechanisms offer a promising paradigm to overcome the limitations of synthetic delivery systems. Recent advances highlight exosomes’ therapeutic potential in EC. Jia et al. (43) identified an exosome DLEU1 as a promoter of EC proliferation, migration, and invasion by regulating the miR-381-3p/E2F3 axis, suggesting the potential use as a therapeutic target for endometrial cancer. To complete this finding, Ding et al. (44) prepared a ultra-pH-sensitive nanovesicle based on poly ethylene glycol—poly (diisopropylamino) ethyI methacrylate (PEG-PDPA) (Figure 4a). The hydrophilic cavity and hydrophobic membrane of this vesicle respectively encapsulate the chemotherapy drug doxorubicin and the anti-apoptotic Bcl-2 inhibitor navixol. The average diameter was 125.0 ± 4.8 nm when the pH value is 7.4. These vesicles achieved tumor-selective drug release in acidic microenvironments, significantly suppressing EC growth *in vivo* with minimal toxicity (Figure 4b).

In addition, some studies explored the role and mechanism of tumor-associated macrophage (TAMs) -derived exosomes in the progression of EC, attempting to identify new pathogenesis and therapeutic targets for endometrial cancer. Wang et al. (45) demonstrated TAMs-derived exosomes inhibit EC cell apoptosis and promote epithelial-mesenchymal transition. This effect was linked to low miR-192-5p expression in TAM exosomes. Similarly, Jing et al. (46) developed exosomes (40–110 nm) delivering miR-499a-5p, which suppresses EC progression by targeting VAV3 to inhibit tumor/endothelial cell proliferation and angiogenesis, offering promise for advanced EC therapy.

Exosomes demonstrate clear advantages for EC treatment. However, translational challenges still exist, such as the poor physiological relevance of 2D cell culture-derived exosomes and discrepancies between animal models and clinical outcomes. Overcoming these hurdles requires advanced *in vitro* models that better recapitulate the tumor microenvironment and rigorous validation in patient-derived systems.

### 4.5 Aptamer

Aptamers are single-stranded DNA or RNA obtained through *in vitro* screening techniques. They exhibit high specificity and affinity toward diverse target molecules, such as proteins, small molecules, and cells. Owing to their exceptional stability, ease of modification, and functional versatility, aptamers hold significant promise as molecular recognition tools and therapeutic agents. Due to their unique molecular recognition ability and engineering convenience, aptamers show potential to be therapeutic tool for compensating for organoid defects. At present, aptamers are widely applied in diagnostics, drug development, therapeutic delivery, and protein function studies.

In the context of endometrial cancer (EC) treatment, doxorubicin remains a standard chemotherapeutic for advanced and recurrent disease. However, its efficacy is limited by inherent tumor cell insensitivity and severe systemic toxicity. While DNA nanotechnology offers substantial potential for oncology applications, its exploration in EC remains limited. Considering that non-receptor tyrosine kinase (SRC) plays an important role in chemotherapy resistance of endometrial cancer, in order to verify that SRC can regulate the sensitivity of endometrial cancer cells to doxorubicin. Li et al. (47) developed a targeted gene-silencing drug delivery platform DSN Clew on rolling ring amplification and bivalent multivalent aptamers (Figure 4c). Studies have confirmed that the constructed DSN Clew platform can precisely target tumor cells, has good biocompatibility and low toxicity, and can enhance the therapeutic effect of doxorubicin on endometrial cancer both *in vitro* and *in vivo*, with lower toxicity than doxorubicin. At the same time, it can enhance the sensitivity of endometrial cancer to ferroptosis through the SRC/STAT3/ACSL4 axis. Thereby improving the reactivity to doxorubicin and achieving a good synergistic treatment. Zhang et al. (48) prepared NP-Apt bioconjugates using biocompatible and biodegradable PLGA-b-PEG copolymer through nano-precipitation method, showing a uniform diameter of 621.5 nm. This platform enabled the simultaneous co-delivery of docetaxel (DTX) and doxorubicin (DOX) at a controlled molar ratio of 9:1, with drug-loading capacity modulated by total drug input (Figure 4d). Studies have confirmed that docetaxel is released relatively slowly, with approximately 50% and 80% released at 6 h and 25 h respectively. Doxorubicin is released relatively quickly, with 50% and 80% released at 4 h and 6 h respectively. The NP-Apt system selectively delivered payloads to PSMA-expressing LNCaP cells, while showing negligible uptake in PSMA-negative PC3 cells, confirming aptamer-mediated targeting precision. Moreover, co-delivery of DTX/DOX significantly enhanced cytotoxicity against LNCaP cells compared to monotherapy, showing the therapeutic advantage of combinatorial loading.

Despite these advances in aptamer-targeted co-delivery, tumor heterogeneity and the dynamic tumor microenvironment remain critical barriers to clinical translation. Future designs must incorporate multiplexed targeting ligands and stimuli-responsive release mechanisms to address spatial-temporal heterogeneity.

### 4.6 Others

**Figure 5:**
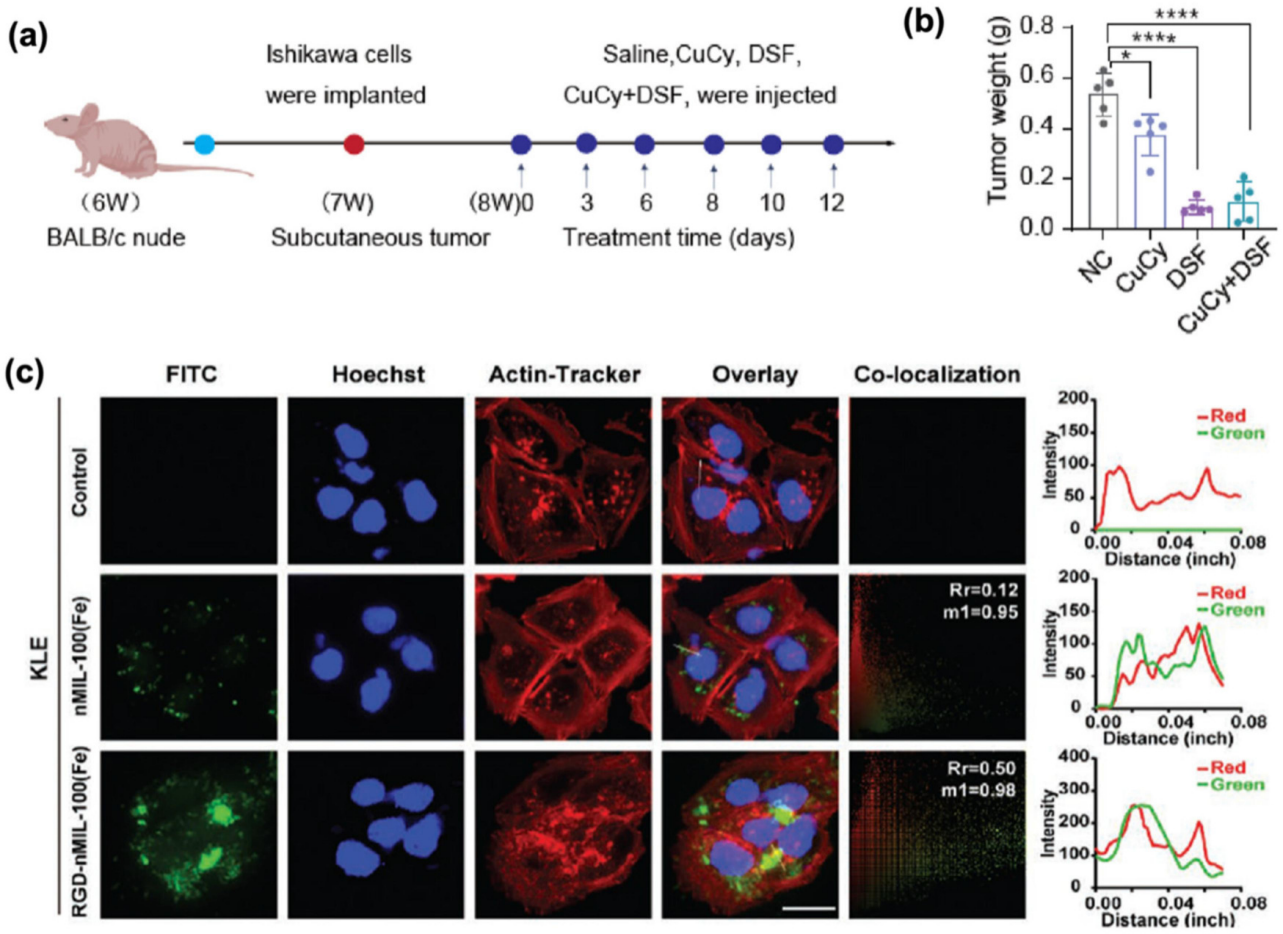
**(a)** Schematic diagram of subcutaneous transplanted tumors and drug administration in nude mice. Adapted with permission (49). Copyright 2024, Bioactive Materials. **(b)** Weight of the transplanted tumors (*n* = 5). Adapted with permission (49). 2024 The Authors. **(c)** Confocal fluorescence images of KLE cells incubated with FITC-labeled nMIL-100(Fe) and RGD-nMIL-100(Fe) nanoparticles for 6 h, displaying nanoparticle internalization (green channel), cell actin (red channel), and nucleus (blue channel) staining (scale bar:10 µm). The Plot Profile plug-in analyzed the colocalization relations; Pearson's coefficient (Rr) and Andersoverlap coefficient (M1) are labeled in the top right corner of the colocalization channels, respectively. Adapted with permission (50). Copyright 2023, Small.

In addition to the nanomaterials mentioned above, repurposed pharmaceutical agents and novel complexes demonstrate significant potential in oncotherapy. Disulfiram (DSF), an established alcohol-aversion therapeutic, exhibits anti-tumor activity when combined with copper. The copper cysteamine (CuCy) complex has photodynamic effects and anti-tumor properties. In view of their advantages, Yang et al. (49) investigated DSF combined with CuCy nanoparticles in the treatment of endometrial cancer. They revealed that low dose (0.5 uM) combination could significantly inhibit tumor growth and angiogenesis and induce apoptosis. The mechanism is that the drug enters the cells through phagocytosis, damages mitochondria, activates the apoptotic pathway, and the safety of combined therapy is superior to that of DSF monotherapy, as shown in Figures 5a,b. Gong et al. (50) have also made breakthroughs in the treatment of type II endometrial cancer (EC) through nano-catalytic medicine (Figure 5c). They engineered an RGD-functionalized nMIL-100(Fe) catalyst (RM) targeting EC cells, which generated cytotoxic reactive oxygen species (ROS), inhibited proliferation, and induced apoptosis. Remarkably, combining RM with the mitochondrial autophagy inhibitor Mdivi-1 and β-lapachone (β-Lap) yielded 85.92% tumor growth suppression and extended survival in mice models.

Instead of transforming the size of traditional medicine to nanoscale, utilizing nanomaterials as the function-integrated theranostic agents is a major approach to reach efficient treatment. Nanomaterials such as iron oxide nanoparticles (51), gold nanoparticles (52), carbon nanotubes (53), and silica nanoparticles (54) can be employed for controlled drug release in tumors therapy (51). For instance, carbon nanotubes can be used as drug delivery systems to carry anti-cancer drugs for targeted release, thereby enhancing therapeutic efficacy and reducing side effects on healthy tissues (55). Similarly, carbon dots, as drug and gene vectors, can achieve controllable and targeted release, reduce toxicity, and further optimize therapeutic effects by enhancing drug solubility and bioavailability and supporting real-time tracking (56). Multifunctional magnetic gold nanomaterials enhance cancer treatment by integrating targeted ligands like antibodies or peptides. This allows for tumor-specific accumulation, concentrating therapeutics at the tumor site while sparing healthy tissues. Furthermore, the photothermal therapy they mediate improves tumor ablation efficiency, and no significant systemic toxicity has been observed (57). In addition, when gold nanoparticles are used as drug carriers, surface functionalization (such as conjugation with folic acid or transferrin) can increase the drug uptake rate of tumor cells, enhance the therapeutic effect. This strategy simultaneously improves therapeutic efficacy and reduces toxicity to healthy cells (58). In immunotherapy, Zandi et al. combined functionalized gold nanoparticles with tumor cell lysates and Freund's adjuvant to treat breast cancer. The results were striking, over 17 days, the treatment group exhibited an 86% reduction in tumor size, whereas tumors in the control group grew by 52%. This represents a dramatic 138% relative improvement in the tumor shrinkage rate, underscoring the platform's dual potential to enhance therapeutic efficacy and minimize toxicity. Such results highlight nanomaterial's dual potential in enhancing therapeutic effects and reducing toxicity, making them promising in cancer treatment. And a number of representative review papers summarized the applications of nanoparticles in cancer therapy (59–62).

Multifunctional and stimuli-responsive nanoparticle systems have shown advantages in prolonging circulation time and enhancing intracellular drug delivery (63). These systems can co-deliver hydrophilic and hydrophobic drugs (48), achieving superior tumor-targeted therapy. The above-mentioned nanomaterials-based system have been well studied in the imaging applications and are candidate nanoplatforms for building up nanoparticle-based theranostics. However, current research remains predominantly exploratory, and more detailed information about the nanoparticle-based theranostics can be found in other review articles (51, 61, 64, 65). Based on such foundations, future studies could focus on designing nanotheranostic platforms specifically tailored for endometrial carcinoma by integrating these advancements with the pathophysiological features of EC. Overall, continued investigation is necessary to develop novel delivery technologies, elucidate resistance mechanisms, and establish precision medicine strategies.

## 5 Conclusions

Endometrial cancer (EC) presents escalating clinical challenges due to rising incidence, limitations of conventional therapies, and the unmet need for fertility preservation in young patients. This review summarized recent advances in nanomaterials-based strategies that offer alternative solutions to the challenges. Due to the unique properties of nanomaterials such as enhanced permeability and retention (EPR), stimuli-responsive drug release, and multifunctional integration, nanoplatforms demonstrate potential to improve therapeutic precision while minimizing systemic toxicity.

Diverse platforms were discussed in this review, including polymeric carriers, quantum dots, liposomes, exosomes, and aptamer-drug conjugates, which enable targeted delivery, controlled release, and synergistic drug combinations, improving efficacy while minimizing systemic toxicity. These platforms provide an alternative approach to address EC treatment barriers: (1) enhanced tumor targeting via EPR/ligand-receptor interactions, (2) stimuli-responsive drug release (pH, enzymes), (3) co-delivery of hydrophilic/hydrophobic agents, and (4) reduced systemic toxicity. They show great potential in overcoming chemoresistance, preserving fertility through non-surgical intrauterine devices, and modulating the tumor microenvironment. Despite promising preclinical outcomes, translational barriers remain, including tumor heterogeneity, scalability issues, safety concerns, and limited clinical data. Future research should focus on multifunctional nanoplatforms, subtype-specific strategies, advanced delivery models, and personalized approaches to bridge the gap from bench to bedside.
